# Effect of Hydrogen Peroxide on Oviposition Site Preference and Egg Hatching of the *Aedes aegypti* (Linnaeus) Mosquito

**DOI:** 10.3390/insects16090928

**Published:** 2025-09-04

**Authors:** Luka Ndungu, Donald Roberts, Lewis Long, Emilie Goguet, Alex Stubner, Sean Beeman, Stephen Lewandowski, Bernard Okech

**Affiliations:** 1 Division of Occupational and Environmental Health Sciences, Department of Preventive Medicine and Biostatistics, Uniformed Services University of the Health Sciences, 4301 Jones Bridge Road, Bethesda, MD 20814, USA; 2 Division of Global Public Health, Department of Preventive Medicine and Biostatistics, Uniformed Services University of the Health Sciences, 4301 Jones Bridge Road, Bethesda, MD 20814, USA; 3 Department of Microbiology and Immunology, Uniformed Services University of the Health Sciences, 4301 Jones Bridge Road, Bethesda, MD 20814, USA; 4 The Henry M. Jackson Foundation for the Advancement of Military Medicine Inc., 6720A Rockledge Drive, Bethesda, MD 20817, USA; 5 Graduate Programs, Department of Preventive Medicine and Biostatistics, Uniformed Services University of the Health Sciences, 4301 Jones Bridge Road, Bethesda, MD 20814, USA

**Keywords:** *Aedes aegypti*, hydrogen peroxide (H_2_O_2_), egg hatch rates, Oviposition Activity Index, oviposition behavior

## Abstract

Hydrogen peroxide (H_2_O_2_) occurs in the environment, including in aquatic environments where mosquitoes might lay eggs. However, little is known about the compound’s impact on mosquitoes. We conducted an experiment to determine the effect of H_2_O_2_ on *Ae. aegypti* oviposition behavior and egg hatching using H_2_O_2_ concentrations similar to those in natural aquatic environments. Oviposition behavior was evaluated by dual-choice and multi-choice bioassays. Gravid *Ae. aegypti* mosquitoes were placed in cages with containers with different H_2_O_2_ concentrations (5, 25, 50, and 100 μM). After 72 h, the number of eggs laid was compared between oviposition sites with and without H_2_O_2_. Additionally, egg hatching was assessed under long-period exposure (48 h) and short-period exposure (2, 4, and 6 h and then in deionized water for up to 48 h). Results showed no significant difference in oviposition preference scores in the multi-choice assay (OAI = −0.135 ± 0.06) (*p* = 0.138), but a significant difference in the dual-choice assay (0.195 ± 0.01) (*p* = 0.001). Long-period exposure to H_2_O_2_ did not significantly affect hatch rates (11.34%) (*p* = 0.363), but short-period exposure significantly impacted hatch rates (17%) (*p* = 0.0001), with period of exposure alone playing a significant role (*p* < 0.0044). Eggs exposed to 100 μM H_2_O_2_ for 2 h (*p* = 0.0070) and 4 h (*p* = 0.0036) had significantly higher hatch rates compared to the control. This study demonstrates that low concentrations of H_2_O_2_ can influence oviposition site characteristics and egg hatch rates. Combined with other environmental factors, H_2_O_2_ can shape the reproductive success of *Ae. aegypti*, offering potential strategies for mosquito control.

## Simple Summary

*Aedes aegypti* (Linnaeus, 1762) mosquitoes, which spread diseases such as dengue and Zika, lay their eggs in water-filled containers in the environment. Hydrogen peroxide is a chemical naturally found in rainwater and other water sources, but its effects on mosquito oviposition choice preference and egg hatching are not well understood. This study tested whether concentrations of hydrogen peroxide (5 to 100 μM) influence where mosquitoes lay their eggs and whether their eggs can still hatch. In experiments offering many choices, mosquitoes laid eggs even in cups with hydrogen peroxide, but when given only two options, water and hydrogen peroxide, they preferred cups without hydrogen peroxide. For egg hatching, long exposure to hydrogen peroxide did not have much effect, but short exposure to higher concentrations increased the number of eggs that hatched. These findings suggest that hydrogen peroxide in the environment can sometimes change mosquito egg-laying behavior and may affect egg hatchability under specific conditions. Understanding these effects can help us predict how natural water chemistry shapes mosquito oviposition behavior and could inform new ways to manage mosquito populations and reduce disease spread.

## 1. Introduction

*Aedes aegypti* (Linnaeus, 1762) productivity in nature is intricately connected to prevailing environmental conditions, such as aquatic habitats, warmer temperatures, and higher humidity levels. Increased mosquito productivity is closely associated with increased presence of aquatic habitats and availability of organic matter that serves as food for larval development [1]. As a major vector of some of the most serious arboviral diseases such as dengue, Zika, chikungunya, and yellow fever, the *Ae. aegypti* mosquito is predicted to expand in geographic range due to climate change, urban heat islands, inadequate solid waste (containers) management, and the rapid pace of urban expansion, among other factors [2,3,4]. These projections highlight the critical need for continued research into ecological factors influencing mosquito productivity, which could guide targeted mosquito control strategies.

*Ae. aegypti* mosquitoes lay eggs and develop in freshwater that has accumulated in artificial and natural containers [5,6]. Among the various factors found in these habitats, such as plant debris, animal detritus, and other decaying organic matter that ends up as dissolved organic matter, cyanobacteria are perhaps the most important as they are highly correlated to the abundance of immature *Ae. aegypti* [7]. Many studies have linked cyanobacteria blooms to H_2_O_2_ hotspots in sub-tropical freshwater ecosystems [8,9,10,11]. Upon measurements, the naturally occurring H_2_O_2_ concentrations in freshwater ecosystems range from nanomolar to micromolar, with the highest recorded levels being 5.3 µM [9,11,12]. The main sources of H_2_O_2_ in these aquatic environments include photochemical reaction with dissolved organic matter [12,13,14,15,16,17] and atmospheric sources [18], with rainwater levels 199.0 µM being the largest contributor of H_2_O_2_ [9,13,18,19,20]. In addition to the aforementioned sources of H_2_O_2_, human application of H_2_O_2_ as an algicide for the control of harmful algal blooms [10,21,22,23] can contribute to its accumulation in various aquatic ecosystems.

Given that mosquitoes widely use these aquatic ecosystems for laying eggs, there is a need to investigate the impact of H_2_O_2_, particularly on oviposition behavior and egg hatchability, as these critical stages might be sensitive to environmental stressors. The findings of this study could provide insights into the reproductive ecology of *Aedes* mosquitoes, offering evidence-based strategies for mosquito management and contributing to the broader goal of reducing the spread of mosquito-borne diseases.

## 2. Materials and Methods

### 2.1. Mosquitoes for Laboratory Assays

*Aedes aegypti* (Linneaus, 1762) eggs (Rockefeller strain) provided by the Walter Reed Army Institute of Research (WRAIR), Silver Spring, MD, USA, were used in the egg hatchability experiments. For oviposition bioassays, *Ae. aegypti* adults were raised in the Uniformed University School of Health Sciences insectary following standard procedures [24]. Briefly, the eggs were hatched by placing them in beakers of deionized water. Twenty-four hours after hatching, larvae were transferred to shallow white plastic rearing trays, placed in an environmental incubator (Powers Scientific, Inc., Pipersville, PA, USA), and fed an appropriate amount of TetraMin baby fish slurry and yeast in a 4:1 ratio to ensure optimal larval health and maintain water quality. Trays were held at 27 ± 2 °C, 80% relative humidity, with a 12:12 h light–dark cycle, in accordance with standard mosquito rearing protocols [24]. After 5–7 days, the pupae were collected from the trays and transferred to mesh cages (BugDorm-1, 30 cm^3^, Megaview Science Education Services Co., Ltd., Taichung, Taiwan) where emerging adult mosquitoes were maintained and later used in oviposition bioassay experiments.

### 2.2. Hydrogen Peroxide Concentrations

The H_2_O_2_ concentrations chosen for experiments ranged from 5 to 100 µM and represent the levels that have been recorded in natural aquatic ecosystems [9,11,12,15]. The H_2_O_2_ solutions were prepared at four concentrations: 5 µM, 25 µM, 50 µM, and 100 µM, by serially diluting a 0.1 mM stock solution made from an approximately 30% w/w H_2_O_2_ solution (Thermo Fisher Scientific, Waltham, MA, USA). Concentrations of the H_2_O_2_ were confirmed using the Pierce™ Quantitative Peroxide Assay Kit (Aqueous) (Thermo Fisher Scientific, Waltham, MA, USA) with absorbance readings at 510 nm being recorded using a Synergy HTX Multimode Reader (Gen 5 software, Biotek, Winooski, VT, USA).

### 2.3. Oviposition Assay Experiments

**Figure 1:**
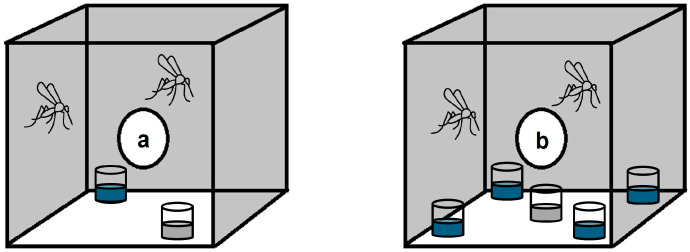
Diagram of the *Ae. aegypti* oviposition bioassay. (**a**) The dual-choice bioassay consisting of two cups and (**b**) the multi-choice assay consisting of five oviposition cups. The control cup is represented by gray color, and the cups with blue color are H_2_O_2_ concentrations. The dual-choice assay was set up in 4 cages, each with H_2_O_2_ (5, 25, 50, and 100 μM), and a control, while the multi-choice assay was set up in a single cage with H_2_O_2_ (5, 25, 50, and 100 μM) and a control.

Adult *Ae. aegypti* were blood-fed and held in cages (BugDorm-1, 30 cm^3^; Megaview Science Education Services Co., Ltd., Taichung, Taiwan) for 3 days until gravid. The gravid *Ae. aegypti* mosquitoes were transferred into the bioassay cages that comprised a multi-choice and dual-choice oviposition setup with an average of two mosquitoes per oviposition cup (Figure 1).

The multi-choice oviposition assay comprised a single cage with five oviposition cups lined with filter paper containing 5, 25, 50, and 100 µM concentrations of H_2_O_2_ and deionized water as a control (Figure 1b). All the cups were placed equidistant from each other, and the dual-choice oviposition assay comprised a total of four cages, each cage with the same configuration of a cup containing 5, 25, 50, or 100 µM H_2_O_2_ and a control cup with deionized water (Figure 1a) that were placed diagonally at opposite corners of the cage. The setup was replicated four times for both the dual-choice and multi-choice bioassays. The gravid mosquitoes were held in the cages, and the cups were inspected after 72 h of egg-laying activity. To recover the eggs, the wet filter papers were carefully removed from the cups, and the eggs were counted under a microscope for each of the four replicates.

### 2.4. Egg Hatching Experiments

*Ae. aegypti* eggs on oviposition papers strips were placed in cups containing H_2_O_2_ concentrations of 5, 25, 50, and 100 µM. The eggs were left under these conditions for a long period and short periods. For a long-period exposure, the eggs were in the containers for 48 h, and hatch rates were assessed at a 48 h time period. For short periods of exposure, the eggs were in the H_2_O_2_ concentrations for 2 h, 4 h, and 6 h and then transferred to containers with distilled water and monitored for 48 h. At the end of the 48 h exposure, the number of hatched eggs was counted for each of the four replicates.

### 2.5. Statistical Analysis

To assess oviposition site preference, the number of eggs laid in each container was used to estimate the Oviposition Activity Index (OAI) [21]. The OAI was calculated using the following equation: OAI = (nH_2_O − nH_2_O_2_)/(nH_2_O + nH_2_O_2_), where nH_2_O is number of eggs laid in water and nH_2_O_2_ is number of eggs laid in H_2_O_2_. An OAI value of negative one (−1) indicated a complete preference for H_2_O_2,_ and a positive one (+1) indicated a complete avoidance of H_2_O_2_ (or preference for water). Zero (0) indicated no oviposition preference for either substrate. To compare OAI values between oviposition sites with and without H_2_O_2_, ANOVA was used for multi-choice oviposition assays, and independent two-tailed *t*-test was used for dual-choice oviposition assays. A paired *t*-test was applied to determine differences in the mean number of eggs laid in H_2_O versus H_2_O_2_ in the multi-choice assay. Descriptive statistics were used to describe the mean and standard error (SE) for OAI scores and mean and 95% confidence intervals (CI) for hatch rates across H_2_O_2_ concentrations. A one-way ANOVA was used to assess the effect of H_2_O_2_ concentrations on hatch rates after a 48 h exposure period (long period). A two-way ANOVA was used to assess the effect of H_2_O_2_ concentrations, the short exposure periods (0, 2, 4, and 6 h), and their interactions on egg hatch rates. Post hoc analysis, using Dunnett’s test, was conducted to identify differences among the concentrations. The analysis used IBM SPSS version 29.0 software, and the threshold for statistical significance was set at *p* < 0.05.

## 3. Results

### 3.1. Effect of Hydrogen Peroxide on Ae. aegypti Oviposition Site Preference

**Figure 2:**
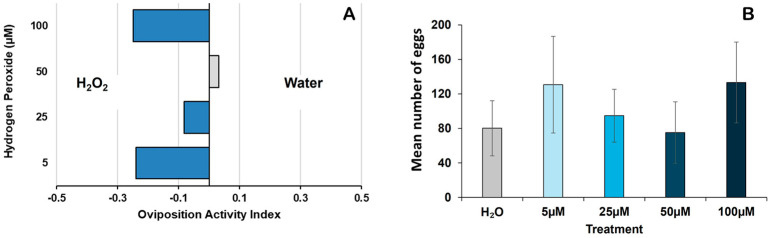
Effect of hydrogen peroxide exposure on *Aedes aegypti* oviposition preference and egg-laying behavior in multi-choice assay (**A**) Oviposition Activity Index (OAI) of *Aedes aegypti* in response to H_2_O_2_ and water at varying concentrations. Negative OAI values indicate preference for H_2_O_2_, and positive OAI values indicate preference for water. (**B**) Mean oviposition (eggs laid) across H_2_O_2_ concentrations (5–100 μM) in a multi-choice assay compared to water. Error bars represent mean SE.

Multi-choice oviposition assay: The mean OAI scores across all H_2_O_2_ concentrations were negative, indicating a preference for H_2_O_2_-treated containers over water (Table 1; Figure 2A). The lowest OAI scores were observed at 5 μM and 100 μM (−0.24 and −0.25), respectively (Table 1). However, no significant differences in OAI scores were detected among the H_2_O_2_ concentrations (*p* = 0.138). Egg counts varied among concentrations, with higher mean egg counts recorded in 5 μM and 100 μM H_2_O_2_ (131 ± 56 and 133 ± 47), respectively (Table 1; Figure 2B). However, these differences in egg counts among H_2_O_2_ concentrations were not statistically significant (ANOVA *p* = 0.775) (Table 1).

**Figure 3:**
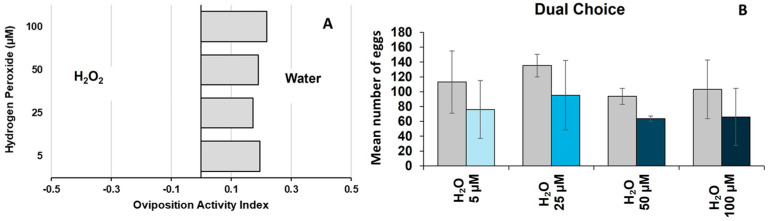
Effect of hydrogen peroxide exposure on *Aedes aegypti* oviposition preference and egg-laying behavior in dual-choice assay (**A**) Oviposition Activity Index (OAI) of *Aedes aegypti* in response to exposure to varying H_2_O_2_ concentrations. Negative OAI values indicate a preference for H_2_O_2_, and positive OAI values indicate a preference for water. (**B**) Mean oviposition (eggs laid) across H_2_O_2_ concentrations (5–100 μM) in a dual-choice assay compared to water. Error bars represent mean SE.

Dual-choice oviposition assay: Based on the OAI scores, the overall mean OAI (across all concentrations) was positive indicating a preference for water over H_2_O_2_-treated containers (Table 1; Figure 3A). Notably, higher positive OAI scores of 0.20 and 0.22 were observed at 5 µM and 100 µM H_2_O_2_ concentrations, respectively (Table 1). Additionally, a significant difference in mean OAI scores was detected between H_2_O and H_2_O_2_ (independent *t*-test; *p* < 0.001). The number of eggs laid in H_2_O_2_ concentrations did not significantly vary (*p* = 0.811). The highest number of eggs was recorded in the control group (111.31 ± 14.11), while the lowest number was observed at 50 µM (63.75 ± 3.57) (Table 1; Figure 3B).

### 3.2. Effect of Hydrogen Peroxide on Egg Hatching

In the long period exposure (48 h), the overall mean hatch rate across all H_2_O_2_ concentrations was 11.35% (95% CI: 8.65–14.04), with the highest hatch rate observed in 5 µM H_2_O_2_. (Table 2).

**Figure 4:**
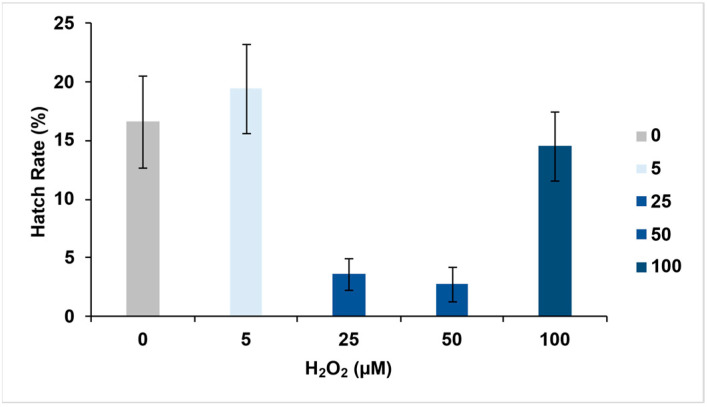
Effect of H_2_O_2_ on hatch rates of *Ae. aegypti* eggs. The figure shows the mean hatch rates for eggs subjected to varying H_2_O_2_ concentrations. Error bars denote adjusted confidence intervals from the mean values from the 4 replicates.

We also observed a decrease in hatch rate at intermediate concentrations and a slight increase in hatch rate at the highest H_2_O_2_ concentrations (Figure 4). However, statistical analysis revealed that these differences were not significant (ANOVA, *p* = 0.363) (Table 3).

**Figure 5:**
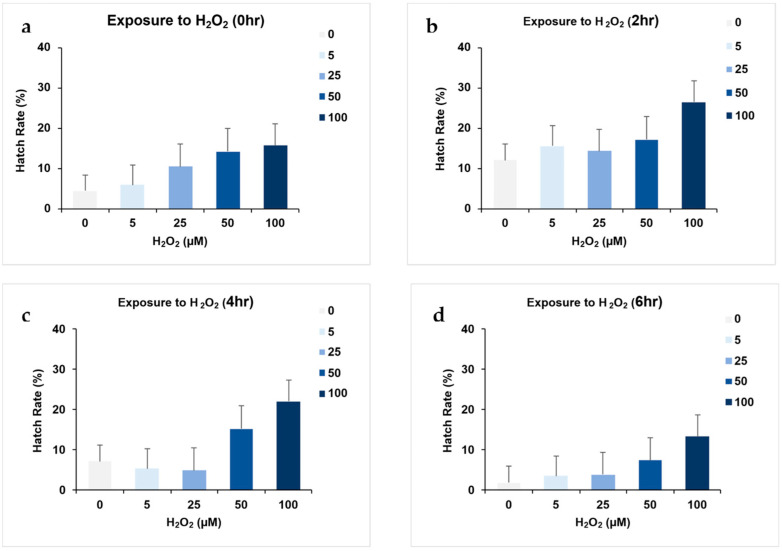
Impact of pre-exposure times on *Ae. aegypti* egg hatch rates at varied H_2_O_2_ concentrations. Panels (**a**–**d**) illustrate the hatch rates following pre-exposure times of 0, 2, 4, and 6 h, respectively, across H_2_O_2_ concentrations. Each bar graph represents the mean proportion of hatch rates derived from two replicates, with error bars indicating the variability around the mean through adjusted confidence intervals.

For the short-term exposure, egg hatch rates increased with H_2_O_2_ concentration across all exposure periods (Table 4 and Figure 5).

In addition, statistical analysis revealed that across all time periods, exposure to H_2_O_2_ concentration significantly affected hatch rates (*p* = 0.0001) (Table 4), and exposure periods also had a significant effect on hatch rates (*p* = 0.0044) (Table 5). No significant interaction was found between H_2_O_2_ concentration and exposure time (*p* = 0.814) (Table 5).

**Figure 6:**
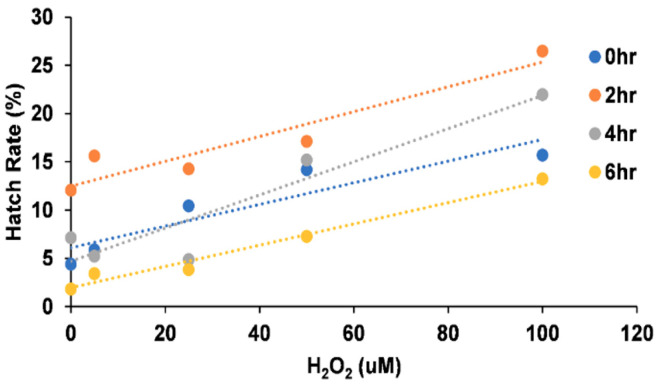
Effect of H_2_O_2_ on *Ae. aegypti* hatch rates across exposure time periods.

Increasing concentration of H_2_O_2_ increased hatch rates across all time periods (Figure 6).

**Figure 7:**
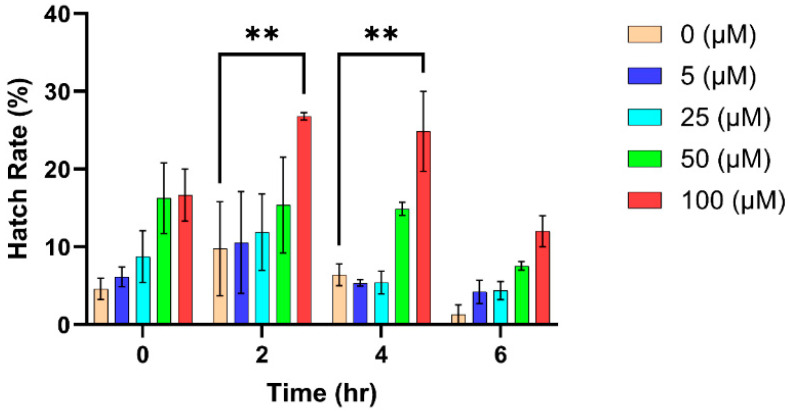
*Aedes aegypti* egg hatch rates (%) at different H_2_O_2_ concentrations (0, 5, 25, 50, and 100 (μM)) measured across time points (0, 2, 4, and 6 h). Error bars represent the 95% confidence interval deviations from the mean. Significant differences between the control (0 (μM)) and the H_2_O_2_ treatments are indicated with asterisks (** *p* < 0.01).

Post hoc analysis by Dunnett’s test revealed a statistically significant difference in hatch rate for eggs exposed to 100 µM H_2_O_2_ compared to the control group for 2 h (*p* = 0.0070) and 4 h (*p* = 0.0036) exposure periods (Table 6; Figure 7).

## 4. Discussion

Environmental factors can shape the distribution and productivity of mosquitoes in nature by influencing their selection of oviposition sites [25,26,27] and the hatching of their eggs [28,29,30]. The data obtained in our study of mosquito oviposition and egg hatching has demonstrated how they might be impacted by low concentrations of H_2_O_2_ found in the environment. It was clear from the results that H_2_O_2_ affected mosquito oviposition site selection but only in the dual-choice oviposition assay. Moreover, hydrogen peroxide affected egg hatching, and the study demonstrated that increasing concentration and exposure time correlated with increased egg hatch rates. Hydrogen peroxide occurs widely in aquatic environments and fluctuates up and down within short time intervals. Therefore, its influence on egg hatching may happen within these short intervals when concentrations are high. Thus, the impact of H_2_O_2_ on egg hatching may complement other environmental factors that are known to influence the hatching of eggs, such as dissolved organic matter. Nonetheless, the mechanisms by which H_2_O_2_ impacts egg hatching and oviposition behavior, both of which are critical aspects of regulating mosquito reproduction, warrant particular attention.

Regarding oviposition behavior, the study demonstrated that H_2_O_2_ can influence oviposition site selection of *Ae. aegypti* mosquitoes, depending on H_2_O_2_ concentrations in those sites and their location relative to freshwater (with no H_2_O_2_). Specifically, when gravid *Ae. aegypti* mosquitoes were presented with multiple oviposition sites with varying concentrations of H_2_O_2_, they laid eggs in all sites regardless of the concentration of H_2_O_2_. However, when presented with a choice of just two oviposition sites, one with H_2_O_2_ and the other without, the mosquitoes frequently selected the container without H_2_O_2_ (freshwater). The seemingly contradictory findings could be explained by the probability that the more available the oviposition sites are, the higher the probability of them being used for oviposition. It is also possible that many oviposition sites in close proximity to each other may disrupt the mosquito’s ability to choose. However, we observed a non-linear response (i.e., a peak-and-trough oviposition pattern) in the multiple-choice experiments, which might indicate that mosquitoes favor a given concentration range of dissolved oxygen in oviposition sites. In natural aquatic ecosystems, H_2_O_2_ concentrations may vary across microscales of space and time, and therefore, mosquitoes’ choice of oviposition sites can be variable over time and space. In the dual-choice experiments, we observed that mosquitoes avoided the containers with H_2_O_2,_ which have high dissolved oxygen and potentially inhibit egg hatching. The presence of H_2_O_2_ might also alter chemical cues and water quality, affecting pH and habitat structure, which could in turn influence mosquitoes’ choice of oviposition sites. It might be possible that the antimicrobial properties of H_2_O_2_ may reduce the composition of nutrients from other microorganisms [31], rendering the sites less attractive because of low nutrient availability for mosquito larval development.

This study observed that *Ae. aegypti* egg hatch rates were positively correlated with concentration of H_2_O_2_. Mosquitoes typically lay their eggs in the photic zones of aquatic ecosystems [27,32] that are characterized by higher levels of dissolved oxygen [33]. Several factors in natural aquatic ecosystems can trigger *Ae. aegypti* egg hatching, including bacteria, organic matter, low dissolved oxygen, and water temperature [34]. However, the increase in hatch rates at elevated H_2_O_2_ levels suggests that H_2_O_2_ itself might play an important role. Although H_2_O_2_ can increase dissolved oxygen levels when it decomposes, high dissolved oxygen is unlikely to cause increased hatch rates. On the contrary, it is low dissolved oxygen that typically induces hatching [35]. In laboratory settings, negative pressure is used to reduce dissolved oxygen to induce hatching of *Ae. aegypti* eggs [24]. In our experiments, it would have been counterproductive to use negative pressure as it would have counteracted the effects of H_2_O_2_, which increases dissolved oxygen. This suggests that H_2_O_2_ may enhance hatchability through mechanisms other than the increasing dissolved oxygen levels. A change in pH caused by H_2_O_2_ would not affect hatching, as previous studies have shown that pH has no impact on hatching [36]. Mosquito eggshells are made of a chitin–protein complex [37,38] and can be rapidly degraded by H_2_O_2_ [39,40]. Therefore, it is likely that egg hatching and larval eclosion would be easier if mosquito eggshells were weakened via degradation by H_2_O_2_. Thus, it is likely that H_2_O_2_ could react with the egg exochorion in ways that promote hatching independently of pH.

Our findings suggest that short-period exposure to H_2_O_2_ may enhance egg hatchability but only at the higher concentration of 100 µM. Lower concentrations of H_2_O_2_ did not appear to significantly affect egg hatchability over either shorter or longer time periods. In our study, we used the 6 h exposure limit as it closely reflects the time it might take for H_2_O_2_ peak production via photochemical action during daylight in the absence of rainfall [16]. The magnitude of the hatching effect of H_2_O_2_ was higher during the time period of 2 and 4 h, suggesting this time range might be ideal for hatching when eggs are exposed to H_2_O_2_. Two to four hours is also nearly the same as the half-life of H_2_O_2_ decay, which ranges from one to seven hours [15,16]. There was no interaction between H_2_O_2_ concentration and exposure time, suggesting that the effects on hatch rates are relatively independent of duration, once the threshold concentration is reached, pointing to an optimal window for H_2_O_2_ exposure needed for hatching. In natural environments, H_2_O_2_ is generated by both abiotic and biotic processes, as previously discussed, and its concentration fluctuates with sunlight intensity [16,41]. Our results suggest that the gradual increase in H_2_O_2_ concentrations during daylight hours, particularly during the crepuscular period when mosquito oviposition typically occurs, may create favorable conditions for egg hatching. During periods of peak sunlight, higher levels of H_2_O_2_ could weaken mosquito eggshells and increase egg hatch rates. Previous studies have demonstrated the importance of reactive oxygen species such as H_2_O_2_ for the hatching success of the eggs of a catfish species (*Ictalurus punctatus*) [42]. High hatch rates were found after *Ae. aegypti* eggs were exposed to very high concentrations of H_2_O_2_ in a BSL-3 laboratory setting as conducted by Hacker [43]. However, this study of Hacker et al. also observed high larval mortality shortly after hatching. Our studies used much lower concentrations and shorter exposure times that did not harm mosquito larva hatchlings. This suggests that H_2_O_2_ may promote hatching at low concentrations in the nanomolar to micromolar range, but very high concentrations in the millimolar to molar range could be harmful or even lethal. Much remains to be understood about the mechanisms by which H_2_O_2_ influences mosquito egg hatching. Future research should explore how H_2_O_2_ affects egg hatching as this could provide key insights into its broader role in mosquito oviposition behavior, with potential applications for population control.

While this study provides valuable insights into the impact of H_2_O_2_ on the oviposition behavior and egg hatching of *Ae. aegypti*, several limitations must be acknowledged. First, the controlled laboratory environment may not fully capture the complexity of natural aquatic habitats, where factors such as temperature fluctuations, organic matter composition, and predator presence could influence mosquito behavior and egg survival. In addition, mosquitoes in nature are free to move about unhindered in their search for oviposition sites, and our use of experimental cages might have confined the *Ae. aegypti* mosquitoes in tight spaces, causing crowding that might have affected oviposition choice. Additionally, the focus on H_2_O_2_ as a single factor does not account for potential interactions with other environmental variables. The concentration range tested, though reflective of natural conditions, may not encompass the full spectrum of H_2_O_2_ variability in ecosystems subjected to anthropogenic influences such as algicide applications. Finally, the findings are specific to *Ae. aegypti*, and further research is needed to determine whether these results are generalizable to other mosquito species. Future field studies are essential to validate these laboratory findings and better understand their ecological implications.

## 5. Conclusions

The study conclusions are that while H_2_O_2_ does not significantly alter the oviposition behavior of *Ae. aegypti* mosquitoes, it does minimally increase the egg hatch rate, particularly at higher concentrations and with extended exposure times. This effect suggests that H_2_O_2_ hotspots in aquatic environments, such as those influenced by sunlight or organic matter, may play a role in enhancing mosquito populations by boosting egg hatch rates. To build on this research, future studies should focus on (1) conducting field investigations to determine how these laboratory findings translate to real-world conditions, particularly in H_2_O_2_-rich environments, and (2) exploring the biochemical pathways through which H_2_O_2_ influences egg hatching. These studies could pave the way for innovative and ecologically sensitive approaches to managing mosquito populations.

## Figures and Tables

**Table 1:** Oviposition Activity Index (OAI) and egg counts for *Ae. aegypti* mosquitoes across two experimental setups: multi-choice and dual-choice oviposition assays, under varying H_2_O_2_ concentrations.

Bioassay	Parameter	Water	5 µM	25 µM	50 µM	100 µM	Overall	*p*-Value
**Multi choice oviposition**	No. eggs	80.25 ± 14.26	130.75 ± 56.06	94.75 ± 30.62	75.25 ± 35.63	133.25 ± 46.83	108.5 ± 20.42	0.775
	OAI	na	−0.24 ± 0.25	−0.09 ± 0.29	0.03 ± 0.32	−0.25 ± 0.24	−0.135 ± 0.06	0.138
**Dual choice oviposition**	No. eggs	111.31 ± 14.11	76 ± 39.11	95.25 ± 46.74	63.75 ± 3.57	66 ± 38.53	75.25 ± 16.46	0.811
	OAI	na	0.24 ± 0.29	0.17 ± 0.30	0.19 ± 0.06	0.22 ± 0.26	0.195 ± 0.01	<0.001 *

Note: OAI (Oviposition Activity Index) values range from −1 (complete preference for H_2_O_2_) to +1 (complete avoidance of H_2_O_2_). No. of eggs refers to the total number of eggs laid at each H_2_O_2_ concentration (µM). Control is deionized water (0 µM). Values are reported as mean ± standard error (SE). Statistical significance (* *p* < 0.05) was determined using ANOVA for multi-choice assay (OAI and egg count) and independent *t*-test for dual-choice assay (OIA and egg count).

**Table 2:** The effect of hydrogen peroxide on the hatch rates of *Aedes aegypti* eggs.

H_2_O_2_ (μM)	Hatched	Total	Hatch Rate (%)	CI Lower	CI Upper
**0**	57	344	16.56	12.64	20.50
**5**	82	423	19.39	15.62	23.15
**25**	25	699	3.58	2.20	4.95
**50**	13	479	2.71	1.26	4.17
**100**	80	552	14.49	11.57	17.43
**Overall Mean**	257	2497	11.35	8.65	14.04

**Table 3:** *Aedes aegypti* egg hatch rates (%) after exposure to varying H_2_O_2_ concentrations for 48 h. ANOVA *P*-value for overall significance.

Hatch Rate	Sum of Squares	df	Mean Square	F	Sig.
Between Groups	558.8	4	139.7	1.171	0.363
Within Groups	1789	15	119.267		
Total	2347.8	19			

**Table 4:** The effect of exposure time and H_2_O_2_ concentration on egg hatch rates of *Aedes aegypti*. The number of hatched and total eggs are sum from all the 3 replicates.

Exposure Period	H_2_O_2_ (μM)	Hatched Eggs	Total Eggs	Hatch Rate (%)
**0 h**	0	19	307	6.19
	5	14	270	5.19
	25	23	278	8.27
	50	139	302	46.03
	100	156	351	44.44
**2 h**	0	145	307	47.23
	5	134	258	51.94
	25	142	302	47.02
	50	151	313	48.24
	100	193	339	56.93
**4 h**	0	181	348	52.01
	5	136	295	46.10
	25	123	308	39.94
	50	150	318	47.17
	100	178	336	52.98
**6 h**	0	28	320	8.8
	5	15	242	6.2
	25	51	345	14.8
	50	56	277	20.2
	100	78	274	28.5

Note: The number of hatched and total eggs are combined total from all the 3 replicates.

**Table 5:** Two-way ANOVA for effects of H_2_O_2_ concentration (µM), time (h), and interactions on *Ae. aegypti* egg hatch rate.

Source	df	Sum of Squares	Mean Square	F-Value	*p*-Value
H_2_O_2_ (µM)	4	1196	298.9	13.02	<0.0001 *
Time (h)	3	411.5	137.2	5.974	0.0044 *
H_2_O_2_ (µM) × Time (h)	12	166.4	13.86	0.603	0.8143

Significant *p*-values indicate the presence of statistically significant differences in hatch rates across different concentrations and time periods, as well as any significant interaction between these two factors. * *p* < 0.05.

**Table 6:** Pairwise comparison of *Aedes aegypti* egg hatch rates across H_2_O_2_ concentrations using Dunnett’s test.

Multiple Comparison	Mean Difference	95% CI	Sig.	*p*-Value
**2 h**				
0 (μM) vs. 5 (μM)	−0.7900	−13.49 to 11.91	ns	0.9994
0 (μM) vs. 25 (μM)	−2.120	−14.82 to 10.58	ns	0.9759
0 (μM) vs. 50 (μM)	−5.623	−18.33 to 7.081	ns	0.5979
0 (μM) vs. 100 (μM)	−17.03	−29.73 to −4.322	**	**0.0070**
**4 h**				
0 (μM) vs. 5 (μM)	1.046	−11.66 to 13.75	ns	0.9983
0 (μM) vs. 25 (μM)	0.9895	−11.71 to 13.69	ns	0.9986
0 (μM) vs. 50 (μM)	−8.485	−21.19 to 4.218	ns	0.2592
0 (μM) vs. 100 (μM)	−18.45	−31.16 to −5.749	**	**0.0036**

Sig. denotes statistical significance, with ‘ns’ indicating not significant (*p* ≥ 0.05) and ** indicating significant differences (*p* < 0.05).

## Data Availability

The data used for analysis can be made available upon reasonable request.
